# Traumatic Neuroses: A Reply to Dr. R. M. Swearingen and the Texas Medical Journal

**Published:** 1894-09

**Authors:** W. B. Outten

**Affiliations:** Dean, and Professor of the Principles and Practice of Surgery at the Beaumont Hospital Medical College, Chief Surgeon of the Missouri Pacific Railway


### TEXAS MEDICAL JOURNAL.

ESTABLISHED JULY, 1885.

PUBLISHED MONTHLY.—SUBSCRIPTION $2.00 A YEAR.

VOL. X. AUSTIN, SEPTEMBER, 1894. NO. 3.

### Original Contributions.

For Texas Medical Journal.

### TRAUMATIC-NEUROSES:—A REPLY TO DR. R. M. SWEARINGEN AND THE TEXAS MEDICAL JOURNAL.

BY W. B. OUTTEN, M. D.,

Dean, and Professor of the Principles and Practice of Surgery at the Beaumont Hospital Medical College, Chief Surgeon of the Missouri Pacific Railway.

IN THE July number of the TEXAS MEDICAL JOURNAL I note an article by Dr. R. M. Swearingen, entitled "A Review of Dr. Wallace and The Railway Surgeons on Spinal Concussions," wherein my name appears, coupled with sharp criticism, and certain assertions, which are doubtless completely satisfactory to the doctor. It is immaterial to me what opinion the doctor may have regarding my character or assertions; there is only one test by which he can prove either of them, and that is, by truthful investigation. We believe with Holmes, that "Truth is tough. It will not break like a bubble at a touch, nay you may kick it about all day like a foot-ball and it will be round and full at evening." Does not Mr. Bryant say, "Truth gets well if she is run over by a locomotive, while error dies of lockjaw if she scratches her finger." My assertions stand open for the doctor to disprove—mere opinion will not verify their truth or falsity. An individual opinion is a very indefinite quantity, and may represent a great deal of knowledge, or, absolute ignorance.

Now, inasmuch as the doctor has quoted me to his own satisfaction, imputed certain actions which he does not know the truth of, I trust that you will grant me the privilege to place my views before the doctor and your readers, that I may appear in my true light. Honest discussion ever yields healthful results; for as Buckle says, "The great enemy of knowledge is not error, but inertness. All that we want is discussion and then we are sure to do well, no matter what our blunders may be. One error conflicts with another, each destroys its opponent, and truth is evolved."

We likewise find your editorial in the same number of the JOURNAL, which says:

"It really begins to look, as intimated by Dr. Swearingen, as if the National Association of Railway Surgeons is to be a kind of kindergarten for the education of witnesses for the future; as if a systematized effort is being, and to be made to cultivate a sentiment adverse to the belief that serious injury to the spinal cord can be inflicted without external and visible lesions, so that in due time railroad surgeons, as experts before courts in damage suits, can honestly and conscientiously swear that there is, and can be, no such thing as railroad spine; they will have been educated to it, by papers and publications of the sort alluded to, and by statistics like those of Dr. Outten, and other methods yet to be developed perhaps, and it can be seen at a glance what valuable witnesses they will be for the defense."

Had Dr. Swearingen or yourself properly studied this subject you would have found that, from investigations up to the present time, the best and most competent authorities deny the existence of "railway spine." Hence, from the doctor's paper and your editorial, the same old idea exists that anything that has the prefix of "railway" to it contains something of a suspicious and dangerous nature; hence the assertion that the National Association of Railway Surgeons are at present engaged in a conspiracy to the detriment of all honest men, to the detriment of an honorable profession, to the detriment of truth. I am free to confess that too much ability is being placed to the credit of the railway surgeon. They can no more disprove truth than any other class of men, and for one I am morally certain that they are neither foolish enough, nor have they the hardihood to attempt such a thing.

I will attempt to show that my own opinions, as regards this subject, have been formed by experience and a study of the most recent writers upon this subject; that my assertions can be backed by the authority of the most prominent authors extant. First, we will quote from Dr. Swearingen, his peculiar ideas and pathology of this condition:

"Is there such a thing as spinal concussion; and are railway accidents, from their nature, more liable to cause them than any other kind of accident? Concussion means commotion, to shake together, and spinal concussion is thus defined: 'The severe agitation, shaking, shock, or general disturbance of the minute parts of the spinal cord. The primary effects of these concussions are probably due to molecular changes in structure; the secondary are of an inflammatory character, dependent on retrogressive changes, such as softening. Four pathological conditions are embraced under the term concussion of the spine: 1st. A jar, disordering to a greater or less degree the cord, without any perceptible lesion. 2nd. Compression of the cord, slowly produced by extravasation of blood. 3rd. Compression of the cord by inflammatory exudations of serum, lymph or pus within the spinal canal; and 4th. Chronic alterations of the structure of the cord itself, as the result of impairment of nutrition,—consequent on the occurrence of one or the other of the preceding pathological states.'"

To any one conversant with the subject, or who will take time to investigate, they will find that the doctor's pathology is antiquated and unverified; that he can not show its verity, nor by a post-mortem case demonstrate the conditions which he presents as being peculiar to "railway spine"; and that there never has been a satisfactory post-mortem made, or demonstration which can prove the assertions which he has above made. He can not show and demonstrate that at the present time there is any such condition as "railway spine," possessing a typical complex of symptoms, and whose lesions are situated in the spinal cord, and resulting from so-called spinal concussion. Again, he can not demonstrate that "railway spine" is in any way peculiar to railways. That traumatic-neurosis occurs upon railways no one denies; that it can and does occur elsewhere is equally true.

It will not be out of place to give a condensed history of the changes as regards the views in pathology from 1866, the date when Erichsen first wrote his book, up to the present time. Probably no subject of recent date in medicine has received more attention and consideration than this condition—traumatic-neurosis, or so-called spinal concussion. For years this condition was described as being peculiar to railways, owing to its frequent occurrence upon them, the serious condition in which the patient was placed, the natural and consequent litigation, the extreme assessment of damages, and its intense subjective character made it a problem of great and unceasing difficulty of solution. Absolute diversity of authoritative medical opinion, its multitudinous symptomatology, and its psychological consequences, make it, at the present writing, one of the great enigmas of medicine. Traumatic-neurosis now includes the narrow appellation of railway brain and spine, since it is a condition that can occur under the most varied circumstances of life. Thorburn, in his excellent work on "A Contribution to the Surgery of the Spinal Cord," says:

"Mr. Erichsen, in a work which, for careful observation and graphic description, is propably unsurpassed in our language, has enumerated the majority of these symptoms, attributing them to a change in or about the spinal cord, frequently to a meningo-myelitis, and classifying them as 'concussion of the spine.' As the result of the views so ably expressed by him, we have the term 'railway spine' now imported into various European languages.

"The most obvious difficulty which arose in the way of this theory was the fact that many, if not all, the symptoms would appear to be of cerebral rather than spinal origin, and Mr. Erichsen's own view of an extension of meningo-myelitis to the cranial contents has appeared to many authors to be unnecessary, arbitrary and unsupported by any evidence; hence Putnam, Walton, Westphal, Moelli, Oppenheim, and many others have looked rather to the brain than to the spinal cord as the source of the evil, and we have the term 'railway brain.' Such a change is, however obviously, but one step towards the truth. 'Railway brain' connotes no definite pathology, and, like its predecessor, includes many diverse conditions."

In the meantime, Charcot having demonstrated the identity of hysteria in the male with that of the female, then became tangible the theories of the Charcot school who claim that hysteria provoked by accident is the source of certain nervous troubles, as paralysis, hyper-æsthesia, anæsthesia and contraction. Later on Charcot still further demonstrated that frequently after traumatism the nervous troubles arising therefrom may produce a condition essentially the same as suggestion can effect in persons capable of being hypnotized. Finally Oppenheim, Strumpel, Knapp, and others, aver that traumatic-neurosis is more of a neurasthenic condition than an hysterical one; that in consequence of its fixedness, tenacity and the combination of peculiar mental states bordering on psychoses, partaking of the nature of melancholias and hypochondrias. Dr. Byron Bramwell draws the distinction between concussion of the spine and concussion of the spinal cord, and states that many of the profession did not know the true nature and significance of this condition. That two separate and distinct meanings were attached to the word "concussion" and that two separate and distinct things which these meanings implied were frequently confounded with each other. In one sense the word "concussion" was used to indicate the mode of injury or violence; in the other the functional or structural change which was produced, or which was supposed to be produced---but this was a very different thing—in the spinal cord by an injury. A case of very severe sprain of the back, due to a fall from a horse, in which for many weeks there was pain and tenderness on percussion and movement, persisted in the back, but in which there was absolutely no nervous symptoms, and drew this conclusion, that it was a fact that such cases just related in which there were developed none of the symptoms of so-called "railway spine;" that these cases were common in practice, and rarely developed any such train of symptoms. But that in the event this individual had received equally as severe concussion of the spine upon a railway, it would undoubtely have been followed by a series of nervous symptoms, which for the sake of brevity might be described as indicative of "railway spine." He showed that where we had falls of stone or coal on the back, as in the case with colliers, that very frequently certain symptoms appeared which were plainly indicative of concussion of the spinal cord, and finally that cases of so-called "railway spine," cases of traumatic-neurosis due to railway accident and injury, were numerous and various, and due to the derangement of the function of the brain, rather than of the spinal cord. The mode of onset, progress and development of symptoms varied with different cases. In some there was evidence of local injury to the back, head or limbs, immediately after the accident, or in the early stages of the case; in others the patient had merely received a general shake up, and there was no evidence of local bruising or local injury. This disproportion between the local injury received at the time of the accident, and the severity and persistency of the subsequent symptoms was highly characteristic and noteworthy. It was also remarkable that the symptoms which we have just described occurred comparatively seldom in railway employes, guards, etc., who had been injured in railway accidents, and that passengers who sustain severe local injuries, such as fractures of the leg, are more or less likely to suffer from them than persons who had been merely shaken up, and were apparently much less severely hurt. He claimed that the symptoms were due in no possible way to organic disease of the spinal cord, the membranes of the spinal cord, or the membranes of the brain, but that they were undoubtedly due to a functional derangement of the brain. It would exceed the limits of this paper to give the assertion of various authors, but suffice it to say that Page, Thorburn, Landon Carter Gray, Strumpell, Oppenheim, Charcot and all recent authorities do not claim the condition as one of spinal concussion, but as being a functional trouble connected with the brain, and not with the spinal cord; the fight being waged as to whether it is of an hysterical or neurasthenic nature.

Concerning the etiology and pathology, we quote from J. A. Booth, M. D., in the *Annual of Universal Medical Sciences*, for 1894, the following:

"Notwithstanding continued imvestigations, the presentation of papers, the reports of cases, no material advance has been made in these directions. It is now the almost unanimous opinion that the results of psychic shock are identical with those of physical shock, and that the psychic element is largely present in the apparently mechanical cases. Charcot has shown that traumatic-neuroses are due, not to the bodily injuries only, but to psychical and physical causes combined."

S. C. Freund points out the fact, that in recent times the French theory has come to be more accepted in Germany, and that Charcot's view is the dominant one. Strumpell believes that traumatic-neurosis is due to "Commotion;" outside of the mechanical disturbances a general predisposition may exist for the hysteria, but generally an external factor is required to produce the result. He regards the condition as equivalent to "traumatic-neurasthenia" and "traumatic hysteria." Jolly prefers the term "traumatic-neurosis" in the singular, and objects to the division into several distinct groups. Schultze objects to all general grouping, and is content to apply to each case following the conditions present the especial designation "neurasthenia," "hysteria," "hypochondria," etc. Schultze believes that direct obliteration of psychical memory pictures of the motions and sensations is produced by the trauma, and assumes that of reflex affection of the brain is produced by a condition of local irritation.

C. H. Wilkinson concludes that it is due to the injury of the sympathetic system of nerves, through the perceptive centers of the brain, and is a true hypochondriasis, kept alive by morbid suggestions and evil forebodings from self and others, as well as by lack of self-confidence, and a neglect of proper exercise, both physical and mental. It is hardly necessary to continue the quotations as regards the etiology and pathology for this trouble, as we conceive that the falsity of Erichsen's position has been proven many years ago. Again, Dr. Swearingen says:

"I am not surprised that the chiefs of railway companies are opening their batteries upon Mr. Erichsen and the so-called railway spine. A careful perusal of the papers read before the National Association of Railway Surgeons since its first meeting will suggest ulterior aims and purposes possibly not suspected by the casual observer. Dr. W. B. Outten, of St. Louis, one of the chiefs, and ex-president, I think, of the association, occupies a high seat in this modern school of surgery, and might be designated as the 'Professor of Theory and Practice." In the *Galveston News* of May 11th, he gives some racy theories on the causes of railway concussion. It is interesting to note how ingeniously he can turn switches, and sidetrack facts that might prove detrimental to railway interests. He says 'the most typical injuries occurring upon the railway occur to the railway employe. His injuries are always far more violent than to the passenger.' 'It can be shown,' continues the doctor, 'that in 22,929 injuries to employes, only eight cases of this trouble (railway spine) occurred among them; or one in 2400; while he can show that one in every sixty-five passengers has this trouble. That when a wreck upon a train occurs near a large city, you will invariably have railway spine, simply for the reason that the neurologist or nerve doctors are always present in the city, while we can show that twenty times the number of accidents occurring upon a road away from a popular center never have them.' "

Now, then, in answer to this, we even make the assertion that we can verify everything we say in connection with it. That about one in every twenty-four hundred injured employes has symptoms which are not always thoroughly and completely developed as traumatic-neurosis, and likewise owing to the peculiar conditions which environ the passenger, about one in every sixty-five, according to the statistics I have kept, complains of some of the symptoms of so-called railway spine. We do not claim that they are true cases of traumatic-neurosis, but we do claim that they settle with the railway company on that basis, and they are aided and assisted by medical and legal advice.

Regarding the fact of this remarkable freedom of railway employes from this trouble, let me assure the doctor the idea is not original with myself, but that many competent authorities have noted this fact and have so stated it. We would refer him first to Thornburn, who says: "Among the large number of railway officials of every source or grade whom I have known, many of whom have been passengers at the time of the collision, but none of whom can claim compensation, I have never met with any who have suffered from severe or persistent nervous symptoms.'' And second, "that in none of the accidents with which I have been concerned, has any railway servant complained of such symptoms. Bruises, fractures, burns and deaths, we meet with only too frequently, but traumatic-hysteria is to me unknown in either of these classes of persons.

As railway officials are similarly constituted to the rest of the population, I presume that they do occasionally suffer from traumatic-hysteria, and I can therefore only conclude that they recover from it within a very brief period.

If the Doctor will take the pains to read Page's work, Bramwell, Gray, and other authorities, he will note that this is likewise commented on.

Pertinent to the question, and well expressed, are the views of Landon Carter Gray. He says:

''Certain it is, that the physical condition of these patients is a very unfortunate one. However uneducated they may be, newspapers and the talk of every day life have filled their minds with dread of the mysterious and baleful consequences that may happen to those who receive injuries, particularly in railway accidents. They have also heard for years of the damages, often enormous, which corporations have been obliged to pay. When the accident occurs the nervous system undoubtedly receives a shock, perhaps intensified by the sight of the killed and wounded, with all the attendant horrors, and this shock should receive immediate and judicious treatment by rest, isolation and medicants. But instead of this a lawyer or his agent, the so-called runner of this country, quickly appears on the scene, and spurs the patient on to a suit for damages, by exaggerating the injury and its consequences, so as to make the too-willing sufferer believe that the company can be readily forced to pay damages. Then come the long years of weary suffering, anxiety, waiting and disappointment, unaided by proper treatment, for, although the patient and the lawyer may not consciously discourage treatment, yet too many hopes and interests would be blasted by a cure, to ever permit of treatment being properly carried on, even if any self-respecting physician could be found for the undertaking. Months, perhaps a year or more, are passed in waiting for the suit to be tried. I have known of three years having elapsed before this was done, because of delay in obtaining evidence; then in fixing the responsibility upon the right corporation; then by alleged corruption of the attorneys, and finally by the long waiting before the case could be reached upon the calendar of the court. When the suit has been brought and pushed to successful determination, and appealed to a higher court, it will usually perhaps be taken to a second higher court, and then months, or years, or more, are passed by.

"In some cases it may even happen that a successful issue in court of highest resort is contested, upon a charge of conspiracy, or some matter of legal technicality that is concocted in order to gain time. Finally, the case being at last successfully ended, it may turn out much more frequently than is dreamed of by those who have not had a long experience, that the costs of the action and the lawyers' fees will leave but a pitiful sum of money at the disposition of the patient. This is not an exaggerated picture by any means; it is a composite portrait of dozens of cases whose details can be taken from my case book. All this disturbance that follows the accident is, oftentimes, I am firmly convinced, a more potent cause of the neurasthenia than the accident itself, and I feel sure that it is the secondary psychical disturbance that makes this form of neurasthenia more intractable than other forms of neurasthenia, although I can not quite agree with the statement which I have often heard made upon the witness stand by very competent colleagues, that traumatic-neurasthenia does not differ from other forms of neurasthenia, except in the physical element. Notwithstanding that Charcot does not exactly say this, it is yet evident that he takes very much the same view in regard to the hysterical origin of these cases; not only, however, are their symptoms to a great extent pathognomonic, but there never yet has been any clinical proof produced to demonstrate the difference between them and other neurasthenic types due alone to mental elements. Nevertheless, I firmly believe that it is the psychical element, together with the resulting lack of proper treatment that makes the usual unfavorable prognosis of this class of cases. In several instances I have persuaded patients to promptly place themselves under treatment, and at the same time, either to abandon or compromise the legal proceedings, and in every instance a cure has been effected. I have a history of fifteen such cases. In other words, in order to make my meaning perfectly plain in a matter of this importance, let me repeat by stating that while I do not believe that traumatic-neurasthenia is a species of disease that has distinctive features of its own, I do not believe that it is of unfavorable prognosis, provided that the psychical element can be excluded, and that prompt and proper treatment can be undertaken. It must be clearly borne in mind, however, that this statement applies only to this group of functional symptoms, and it does not apply to organic diseases caused by injury. But moral responsibility can not be hereby avoided by the persons or corporations by whom the injury has been caused, for whether the neurasthenia is rendered more intractable or not by accompanying psychical element, the injury itself has been the cause of all the symptoms; whether the legal responsibility of these individuals or corporations is lessened on this account is a question of law for the courts to determine, and we physicians have nothing whatever to do with this aspect of the question."

My own conclusions concerning this subject may be summe up briefly as follows: I do not believe that there is any sharply defined type of disease which can be properly called traumatic-neurosis, but I believe that there are many cases which are dependent entirely upon the force and conditions which are created after the physical injury. While realizing that owing to the by-play of extraordinary circumstances trivial injuries, particularly with back symptoms, can readily and easily shape themselves into neuroses, we are firm in the belief that racial differences, perfection of physical condition, and the multiplex force of surroundings, are constantly aiding and abetting factors. I believe that favorable surroundings, the influence of an unprejudiced and thoroughly impartial surroundings, the influence of an unprejudiced and thoroughly impartial medical attendant will create, *cœteris paribus*, the exact condition of the trauma. And if the trauma produces a condition similar to hypnosis, I cannot help but believe that the existing forcible and predetermined mental condition of the medical attendant will often force trivial conditions into serious ones. We believe that many a case of traumatic-neurosis has been treated by persons unacquainted with it, and cured, simply for the reason that they have not been filled with suggestion; it would seem rather startling that the physician was competent by his intense mentality and absolute force of suggestion to create an essentially serious condition, but I candidly believe that there is, in that unknown mental influence, when exerted upon a weakened and morbidly receptive mind, a power competent to suggest, maintain and develop sequences which are serious in the extreme. There cannot be any doubt but what, if I may so use the word, "traumatized" minds absorb sensation, not only direct from the effects of injury, but they take and receive impressions produced by suggestions of an already convinced mind. If hypnosis has been used as a means of treatment in these cases, and success has occurred, it certainly seems natural that along with the conditions existing it would be competent to develop such cases. Again, we maintain that where we have physical perfections, mental strength, and proper knowledge, trauma is very rarely likely to produce the psychic conditions as developed in traumatic-neurosis. For we find that where intelligence is blunted, where surroundings have made incidental elements of danger familiar and not impressionable, physical injuries are manifested as physical injuries, and very rarely terminate in psychic troubles, unless there is intensity of conditions, a predisposition upon the part of the individual, be it either racial, predisposing or acquired.

Doctor Swearingen again says: "When we remember that railway surgeons, as a rule, are the only ones to attend to the injured employe, it is remarkable that a single case is found of that most obnoxious of all diseases. The probabilities are that those eight cases must have, in some inscrutable manner, escaped from railway hospital, and been treated by unpretentious, old fashioned practitioners."

Now, then, we will make this assertion to the doctor, that we doubt the ability of the average medical practitioner to make a prompt and proper diagnosis of a case of traumatic-neurosis; for we claim that absolutely a special training is required for their elucidation, and that the neurologist, by his experience and special training, is the competent one to elucidate them. How many physicians in general practice are competent to interpret the multiplicity of symptoms in traumatic neurasthenia or hysteria; determine the anæsthesia, the involvement of the organs of sense, to determine hemi-anæsthesia, the various hyperæsthesiæs and paræsthesiæs? How many ordinary practitioners are there who are competent to determine if there is contraction of the visual field, the presence of achromatopsia, etc., the various conditions of the reflexes, mental elements, etc.? It is not detracting from the merit of the average practitioner when you say this, because it is plainly known by all conversant with the subject, that a special training is necessary for their determination. The doctor says:

"With the lights before us, it is now safe to assume that had either one of the eight cases been entered on the hospital book within the last year we could find opposite their names either 'traumatic-psychosis' or 'hypochondriasis.'"

I desire to say that the St. Louis Hospital of the Missouri Pacific Railway has a consulting staff, composed of such eminent specialists as Dr. A. Alt, Dr. Robt. Barclay, Dr. J. K. Bauduy, Dr. Keeber, Drs. Mulhall and Loed, and others. We believe most of these gentlemen have a national reputation, and are honest and competent.

Now, as regards these eight cases which the doctor singles out, I will say that only six of these were treated in the St. Louis Hospital, and that in every instance the consulting staff were requested to see and treat them. The writer was eager to get the views of the consulting staff, and every one of these cases was examined by such physicians as Hughes, Shaw, Bauduy, and others. In one particular case, Drs. Hughes and Shaw, Mulhall and Alt, rendered their assistance to the writer in the solution of the diagnosis.

Finally, let me say this: every statement I have made I can verify; now let the doctor disprove them. I claim to be honest in my views, and if they are false, they certainly will be disproved. I claim still further that a student of medicine, true to his calling as a physician, has neither time nor inclination to warp truth, nor is he likely to be foolish enough to imagine for an instant that anything else other than truth will prevail. I care not from what source truth may come, I will accept it with pleasure, and any truth that Dr. Swearingen, or any other confrere will anuounce to me, will be accepted and defended.

